# Alternative splicing in cardiomyopathy

**DOI:** 10.1007/s12551-018-0439-y

**Published:** 2018-07-26

**Authors:** A. Beqqali

**Affiliations:** Centre for Cardiovascular Science, Queen’s Medical Research Institute, University of Edinburgh, 47 Little France Crescent, Edinburgh, EH16 4TJ UK

**Keywords:** Alternative splicing, Heart failure, Cardiomyopathy, RBM20, circRNAs, lncRNA

## Abstract

Alternative splicing is an important mechanism used by the cell to generate greater transcriptomic and proteomic diversity from the genome. In the heart, alternative splicing is increasingly being recognised as an important layer of post-transcriptional gene regulation. Driven by rapidly evolving technologies in next-generation sequencing, alternative splicing has emerged as a crucial process governing complex biological processes during cardiac development and disease. The recent identification of several cardiac splice factors, such as RNA-binding motif protein 20 and 24, not only provided important insight into the mechanisms underlying alternative splicing but also revealed how these splicing factors impact functional properties of the heart. Here, we review our current knowledge of alternative splicing in the heart, with a particular focus on the factors controlling cardiac alternative splicing and their role in cardiomyopathies and subsequent heart failure.

## Introduction

The heart exhibits adaptive responses to a wide array of genetic and external factors, such as hypertension, to maintain contractile function. When compensatory responses are not sustainable, cardiac dysfunction occurs, leading to heart failure where the heart is unable to pump enough blood through to meet the body’s needs for nutrients and oxygen. The failing heart undergoes several structural alterations, most notably hypertrophy of cardiomyocytes, dilation of the ventricles, an increase in extracellular matrix proteins, and potentially also cell death. Heart failure is an increasingly prevalent and lethal disease that is often caused by underlying cardiomyopathies.

Cardiomyopathies are a heterogenous group of disorders where the structure and function of the heart is affected. They either are confined to the heart or are part of systemic disorders. Cardiomyopathies can broadly be categorised as dilated cardiomyopathy (DCM), hypertrophic cardiomyopathy (HCM), restrictive cardiomyopathy (RCM), ischemic cardiomyopathy (ICM), and arrhythmogenic right ventricular cardiomyopathy (ARVCM) (Elliott et al. 2008; Muchtar et al. 2017; Pinto et al. 2016). Cardiomyopathies where no pathogenesis can be identified are generally termed idiopathic cardiomyopathy (Braunwald 2017). The most common form of cardiomyopathy is DCM with HCM as a close second.

The European Society of Cardiology has defined DCM as dilation of the left or both ventricles that is not explained by abnormal loading conditions or coronary artery disease (Elliott et al. 2008). DCM is characterised by increased ventricular diameter with ventricular walls of approximately normal thickness and varying extents of fibrosis. Classification guidelines indicate that DCM may be diagnosed when coronary artery disease, valvular disease, abnormal loading conditions, hypertension, and congenital heart disease are ruled out as primary cause of cardiac dysfunction (Elliott et al. 2008).

The prevalence of DCM and of familial DCM is not fully known, but is believed to be underestimated (Hershberger et al. 2013). DCM is the most common cause of cardiac transplantation and death for non-ischaemic heart failure in young adolescents and adults (McNally and Mestroni 2017; Taylor et al. 2006) and the reported incidence rate ranges from 1:2700 (Codd et al. 1989) to 1:250 (Hershberger et al. 2013). Up to half of DCM cases are familial and causative mutations have been described in more than 50 genes encoding mostly structural components of cardiomyocytes directly involved in the cardiac contractile machinery (McNally and Mestroni 2017). However, a novel molecular mechanism of heart disease has emerged in the past decade that is not directly involved in the contractile machinery of the heart. Driven by rapidly evolving technologies in microarray and next-generation sequencing, aberrant RNA splicing has emerged as a mechanism associated with cardiomyopathies (Kong et al. 2010; Lee et al. 2011; Song et al. 2012).

In this review, we will discuss the importance of alternative splicing in the heart and individual components of the splicing machinery that have been identified in recent years to cause cardiomyopathy. In addition, we discuss possible therapeutic interventions and future directions of research.

## Alternative splicing

**Fig. 1:**
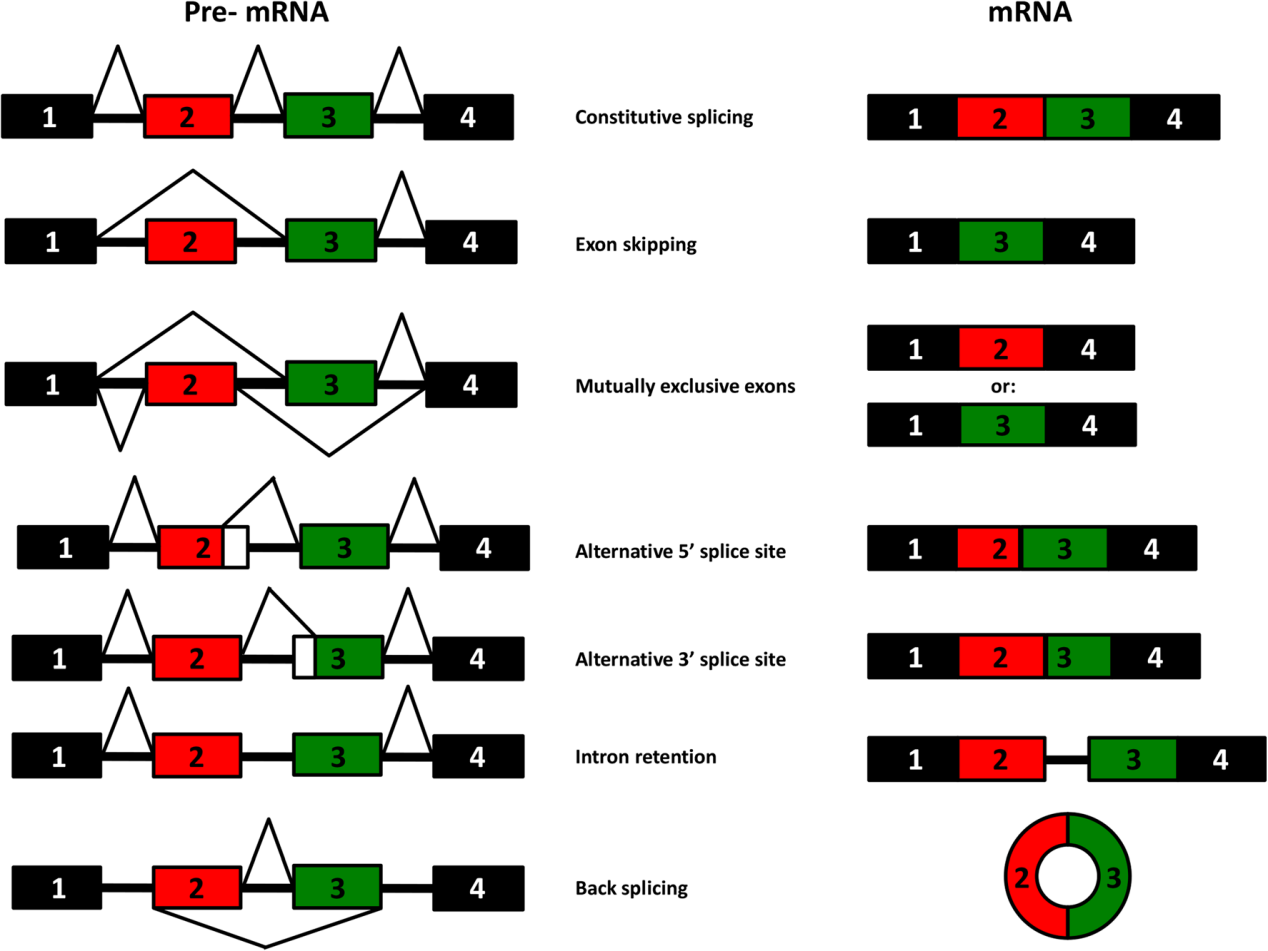
Different processes of alternative splicing

RNA splicing is the molecular process by which introns are removed from precursor RNAs and exons are linked together to form the mature mRNA. This process, which occurs mainly in the nucleus, can be broadly divided into constitutive splicing and alternative splicing. Constitutive splicing is considered the default pathway whereby all introns are removed from pre-mRNA and exons are joined together in the same order as transcribed from the genome. On the other hand, alternative splicing results in exons that can be in- or excluded in different combinations to create a diverse array of functional RNA transcripts from a single gene (Fig. 1).

Nearly all human multi-exon genes undergo alternative splicing, indicating that this post-transcriptional step is central for human gene expression. Unlike promoter activity that is predominantly reflected in the abundance of transcripts, alternative splicing influences the structure of the mRNAs and their potential encoded proteins. As a result, it influences binding properties, intracellular localization, enzymatic activity, protein stability, and post-translational modification of numerous gene products (Manning and Cooper 2017).

Yang and colleagues (Yang et al. 2016) demonstrated that changes in alternative splicing have a large impact on protein-protein interaction partners. Alternatively spliced isoforms of proteins exhibit strikingly different interaction profiles and thus, in the context of global interactome networks, appear to behave as if encoded by distinct genes rather than as minor variants of each other (Yang et al. 2016). Alternative splicing is therefore a post-transcriptional mechanism to generate protein diversity from individual genes, which greatly expands the functional abilities of cells.

The most common type of alternative splicing consists of a single cassette exon that is either included or skipped in the mature mRNA (Kim et al. 2008). Cassette exons can also be spliced or skipped in tandem or spliced in a mutually exclusive manner as shown in Fig. 1. Another form of alternative splicing is intron retention whereby (part of) introns are retained in the mature mRNA which are either translated or end up in the non-sense-mediated decay pathway (Vanichkina et al. 2018). Alternative 5′ or 3′ splice site selection results in short and long forms of an exon, thereby creating alternative open reading frames, that when translated, result in different protein isoforms. Lastly, back-splicing has emerged as relatively new category of alternative splicing which results in the formation of circular RNAs (circRNAs). Nigro and colleagues first described them in 1991; however, this species of RNA molecules was largely ignored due to their unusual splicing behaviour in which exons are joined at consensus splice sites, but in a shuffled order relative to the primary transcript (Nigro et al. 1991). Only decades later with the evolution of next-generation sequencing, the vast expression of circRNAs became evident (Jakobi et al. 2016; Jeck and Sharpless 2014; Khan et al. 2016; Memczak et al. 2013; Salzman et al. 2012; Tan et al. 2017; Werfel et al. 2016).

The first functional studies on circRNAs revealed a possible role in gene expression regulation. Circular RNAs can act as efficient miRNA antagonists (microRNA “sponges”) (Hansen et al. 2013; Memczak et al. 2013; Zheng et al. 2016), whereby the circRNA harbours dozens of highly conserved sequences that can efficiently bind specific microRNAs and thereby strongly suppress microRNA activity. CircRNAs have also been shown to facilitate transcription of their host gene by directly associating with RNA polymerase II (Zhang et al. 2013) or form platforms for protein interactions (Du et al. 2016). Interestingly, emerging evidence suggests that some circRNAs contain open reading frames that can be translated into proteins (Legnini et al. 2017; Pamudurti et al. 2017; Yang et al. 2017).

Splicing is carried out by the spliceosome, a large ribonucleoprotein (RNP) complex found primarily within the splicing speckles of the cell nucleus. The spliceosome is comprised of more than a hundred core proteins (Jurica and Moore 2003) and five small nuclear RNAs (snRNAs U1, U2, U4, U5, and U6). The core splicing signal in precursor RNAs includes three elements that are present in every intron: the 5′ splice site (which includes the GU nucleotides), the 3′ splice site (which includes the AG nucleotides and the polypyrimidine tract), and the branch point sequence (Wang and Burge 2008).

**Fig. 2:**
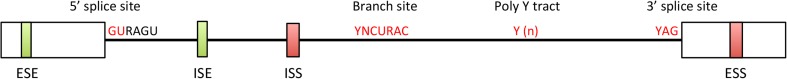
Cis-regulatory sequences necessary for splicing. The four basic splicing sequences are located in the 5′ splice donor site, the 3′ splice acceptor site, the branchpoint sequence, and the polypyrimidine tract (poly Y tract). RNA-binding proteins of the spliceosome bind to these sequences and catalyse the splicing reaction. Exonic and intronic splicing enhancers and silencers (ESE, ISE, ESS, ESI) determine the efficiency of exon inclusion. The branchpoint sequence is located approximately 30 bp upstream of the 3′ splice site while the poly Y tract is located between the branch point sequence and the 3′ splice site. (N, any nucleotide; Y, C/U; R, A/G)

Alternative splicing regulation is mediated by *cis*-regulatory sequences found in the exon and in neighbouring introns. *Cis*-regulatory sequences can facilitate inclusion or exclusion of an exon by recruiting RNA-binding proteins that bind the RNA molecule and act as trans-regulatory factors (House and Lynch 2008). Exonic splicing enhancer (ESE) and intronic splicing enhancers (ISE) recruit splice factors that subsequently facilitate the inclusion of an exon in the mature transcript. While exonic splicing silencers (ESS) and intronic splicing silencers (ISS) facilitate the exclusion of an exon in the mature transcript (Fig. 2).

In general, splicing enhancers bind Ser/Arg-rich domain-containing splice factors (SR proteins), which facilitate spliceosome assembly, whereas splicing silencers recruit proteins of the hnRNP family, which can interfere with recruitment of the spliceosome or SR proteins. SR proteins are characterised by the presence of at least one RNA recognition motif (RRM) and a serine/arginine-rich domain (RS domain). The RNA recognition motif domain is required for RNA-binding, whereas the RS domain functions as a protein interaction domain.

However, it has been shown that these splice factors can have a dual role as splicing enhancer or repressor depending on the context (Sun et al. 2012; Wang et al. 2012; Zhang et al. 2010). This highly complex splicing machinery and in-depth molecular mechanisms of alternative splicing are reviewed elsewhere (House and Lynch 2008; Lee and Rio 2015; Wahl et al. 2009; Wang and Burge 2008; Will and Luhrmann 2011).

## Alternative splicing in the heart

### Alternative splicing in heart development

In addition to its central role in increasing transcriptome complexity and proteomic diversity, alternative splicing also drives decisive physiological changes. The physiological changes that occur before and after birth are critical as the foetal heart adapts to birth and converts to adult function to meet the demands of increased workload in the developing organism (Olson 2006). These developmental and postnatal changes are accomplished through transcriptional and post-transcriptional networks, including alternative splicing.

The importance of alternative splicing in the heart has been pioneered by the study of individual developmentally regulated splice events in genes such as cardiac troponin T (cTnT) (Cooper and Ordahl 1985). In the embryonic heart, exon 5 of cTnT is predominantly included in mRNAs but is excluded in cTnT mRNAs expressed in the adult heart (Cooper and Ordahl 1985). Exon 5 encodes a ten amino acid protein domain, which makes embryonic cTnT-containing myofibrils more sensitive to calcium than adult cTnT myofibrils and thereby influences the contractile properties of embryonic myocardium (Godt et al. 1993; McAuliffe et al. 1990).

Other critical genes in the heart such as myomesin (Myom1) (Schoenauer et al. 2011), titin (ttn) (Lahmers et al. 2004), and LIM domain-binding 3 (Ldb3) (Huang et al. 2003) have been shown to have developmentally regulated isoforms with distinct functions.

The extent of developmentally regulated alternative splicing became clear when Kalsotra and colleagues (Kalsotra et al. 2008) were the first to study transcriptome-wide changes in alternative splicing during heart development using exon arrays. The study revealed 63 alternative splicing events, which were associated with enriched motifs for the splicing factors CUGBP Elav-like family member (CELF) and muscle blind-like splicing regulator (MBNL). While CELF proteins decrease during cardiac development, MBNL increases. Manipulation of CELF and MBNL expression in the adult heart to replicate their levels in the embryo results in reactivation of the embryonic splicing pattern. In a later study, the same group demonstrated that the developmental downregulation of CELF proteins CUG-binding protein 1 and 2 (CUGBP1 and 2) in the heart is mediated by microRNAs (Kalsotra et al. 2010). In addition, a large-scale RNA sequencing study revealed that alternative splicing transitions occur during late embryonic and postnatal mouse heart development, and demonstrated that protein isoform switches are important regulatory components of postnatal cardiac development (Giudice et al. 2014). Altogether, they identified a highly conserved and highly regulated programme of alternative splicing that supports postnatal growth and maturation of the developing mouse heart.

In a more recent study, Wang and colleagues performed genome-wide profiling of alternative splicing transitions between human foetal and adult hearts for the first time using RNA-seq data (Wang et al. 2016). The difference in alternative splicing was mainly observed in protein-coding genes rather than in long non-coding RNAs. Interestingly, intron retention occurred more frequently in the foetal hearts than in the adult hearts, indicating that intron retention may be involved in human heart development. The foetal- and adult-specific alternative splicing events were enriched in mainly cell proliferation functions and energy-specific categories, respectively. Such splicing transitions during human heart development have also been observed in mouse and chicken heart development (Giudice et al. 2014).

### Alternative splicing in heart failure and cardiomyopathy

Genes that are important for cardiac function can be mis-spliced in heart disease (Anderson et al. 1991; Neagoe et al. 2002; Schoenauer et al. 2011), but the extent of mis-splicing has only become clear in the past decade with the technological advances in microarrays and RNA sequencing. It has now been established that altered splicing contributes to a large number of human disease (Scotti and Swanson 2016).

In cardiomyopathy, abnormal splicing of sarcomeric and ion channel genes has been reported in several studies. These changes can ultimately alter the normal internal architecture and homeostasis of the heart leading to heart failure (Lara-Pezzi et al. 2013; Noyes et al. 2017; van den Hoogenhof et al. 2016; Zhu et al. 2017).

Kong and colleagues demonstrated for the first time that alternative splicing is broadly altered in human heart failure (Kong et al. 2010). Using exon arrays, they evaluated RNA splicing in left ventricles of patients with ischemic cardiomyopathy compared to control left ventricles. This revealed aberrant splicing of several sarcomere genes such as cardiac troponin T, cardiac troponin I, filamin C, and β-myosin heavy chain, which have all been implicated in cardiomyopathies before. Next to ischemic cardiomyopathy, these splicing events could be confirmed with RT-PCR in dilated cardiomyopathy and aortic stenosis left ventricular tissue. Interestingly, the splicing changes preceded the onset of heart failure in aortic stenosis samples, which is often accompanied by left ventricular hypertrophy. Furthermore, the authors demonstrated that the identified mRNA splicing patterns accurately classified samples by diagnostic label, providing proof of concept that mRNA splicing profiles may have utility as diagnostic or prognostic markers in heart disease (Kong et al. 2010).

Previous studies have shown that the foetal cardiac gene programme is reactivated in cardiac hypertrophy induced by pressure overload (Barry et al. 2008; Olson 2006; Rajabi et al. 2007). These genes typically play roles in metabolic and contractile functions of the heart and are regulated by a set of transcription factors, which play critical roles in heart development (Oka et al. 2007; Taegtmeyer et al. 2010). These findings led to the question whether the reactivation of a foetal gene programme in cardiac hypertrophy also involves a “foetal RNA splicing” programme.

Park and colleagues where the first to perform a systematic genome-wide approach to systematically define gene expression and alternative splicing profiles in cardiac hypertrophy in comparison with embryonic and postnatal stages of heart development in the mouse (Park et al. 2011).

They found that cardiac hypertrophy induced by transverse aortic constriction involves widespread mRNA isoform changes. While some isoform changes were hypertrophy-specific, other events were associated with development, particularly for the events regulated at the early stage of hypertrophy, suggesting activation of a foetal post-transcriptional programme in the heart in response to pressure overload. Gene Ontology analysis indicated that regulated alternative splicing events are biased to genes with functions in cell adhesion and cell morphology, suggesting an important role of alternative splicing in remodelling the heart. Their analysis also indicated that downregulated expression of Forkhead box protein 1 (Fox-1) during cardiac hypertrophy may play a role in establishing the foetal splicing programme in the hypertrophied heart. This suggests that mRNA isoform regulation plays critical roles in remodelling the heart under pressure overload. The concept that hypertrophy is characterised with re-expression of a foetal splice variant programme was later confirmed by Ames and colleagues in a rat model of cardiac hypertrophy (Ames et al. 2013). Interestingly, almost half of the observed alternative splice variants in hypertrophy were normally expressed in the foetal heart. These findings suggest that cardiac hypertrophy shares post-transcriptional as well as transcriptional regulatory mechanisms with foetal heart development.

Cardiac hypertrophy is generally categorised in physiological hypertrophy and pathological hypertrophy. Physiological hypertrophy is activated by exercise training and can lead to increase cardiac size that is characterised by normal cardiac morphology with a normal and/or enhanced cardiac function (Ooi et al. 2014). Pathological hypertrophy compensates for increased workload; however, its progression generally leads to adverse cardiac remodelling and cardiac dysfunction often leading to heart failure. The underlying molecular mechanisms responsible for the different types of hypertrophic adaptations remain unclear. In an attempt to elucidate some of the molecular mechanism differentiating pathological hypertrophy from physiological hypertrophy, Song et al. performed deep RNA sequencing on mouse models of pathological and physiological hypertrophy of the heart (Song et al. 2012). They found 513 exons to be differentially expressed in pathological hypertrophy, while 414 exons were differentially expressed in physiological hypertrophy. The changes in alternative splicing were mostly related to gains or losses of functional domains, changes in activity, and localization of the encoded proteins. Further bioinformatics analysis of the differentially spliced genes revealed that the signalling pathways involved in physiological hypertrophy were strikingly different from pathological hypertrophy. The identification of highly specific transcriptomic signatures related to physiological and pathological hypertrophy respectively could provide useful insights into understanding the mechanisms underlying both conditions.

To gain more insight into the *cis*- and *trans*-regulatory factors involved in pressure-overloaded cardiac hypertrophy, the same group employed a systematic approach to identify cis-regulatory elements in differentially spliced genes of their previously published RNA-seq data set. Bioinformatics analysis revealed binding motifs in the intronic regions involved in exon exclusion and inclusion, which predicted the binding of splicing factors such as muscleblind-like (MBNL), splicing component 35 kDa (SC35), serine/arginine-rich splicing factor 1 (SRSF1), epithelial splicing regulatory protein (ESRP), polypyrimidine tract binding protein (PTB), and CUG-binding protein 2 (CUGBP2). They could experimentally confirm that protein levels of a subset of these predicted splicing factors were significantly altered during cardiac hypertrophy. This suggests that chronic pressure-overloaded hypertrophy is closely associated with distinct alternative splicing due to altered expression of splicing factors (Kim et al. 2014).

To date, most transcriptome-wide alternative splicing studies have been performed on mouse models of cardiac disease. To establish whether the alternative splicing profiles discovered in mouse models are conserved in humans, more studies are needed on clinically relevant heart samples.

Recently, a large-scale RNA sequencing study on hearts of 97 patients with dilated cardiomyopathy and 108 non-diseased controls revealed 1212 exons that were significantly different between DCM patients and donor control hearts (Heinig et al. 2017). Of the 899 differentially spliced genes, 11 were established genes implicated in DCM. Furthermore, the differentially spliced genes were enriched for the GO terms “MAPK binding”, “actin filament organisation”, “Z disc”, and “I band”. This suggests that most alternative splicing changes were affecting the contractile machinery of the cardiomyocyte, thereby contributing to the DCM phenotype. However, whether the splicing profile resembled that of foetal stages was not investigated. Interestingly, utilising a combination of genotype SNP arrays and RNA sequencing on each sample revealed an important role for genetic variation in determining RNA splicing profiles (Heinig et al. 2017). This suggests that RNA splicing differences in dilated cardiomyopathy are in part controlled by genetic factors.

Altogether, the transcriptome-wide studies of the past decade established a strong association of mis-splicing of critical cardiac genes with hypertrophy, dilated cardiomyopathy, and heart failure. Whether these widespread changes have a significant contribution in disease onset or progression to heart failure is not clear. Therefore, it is important to understand the regulation of alternative splicing that is largely mediated through RNA-binding proteins.

## Splicing factors implicated in cardiomyopathy

### Mouse models of splicing factor-related cardiomyopathy

Myocardial expression of many RNA-binding proteins changes in heart failure, both in human patients and in mouse models, following a general downregulation of splicing-related factors (Felkin et al. 2011; Kong et al. 2010; Park et al. 2011). This suggests that downregulation of splice factors in the heart could have a major role in the aetiology of disease. Indeed, cardiac-specific knockout of a splicing factor has been shown for the first time to cause DCM by Ding and colleagues (Ding et al. 2004). Cardiomyocyte-specific knockout of the SR splicing factor splicing component 35 kDa (SC35) led to the development of DCM around 5 weeks of birth. The same group demonstrated a year later that cardiomyocyte-specific knockout of another SR protein family member alternative splicing factor 2 (ASF/SF2) (also known as SRSF1) results in the development of DCM by week 6 after birth and rapidly progression in heart failure, where mice die around week 8 (Xu et al. 2005). The authors identified a subset of functionally important genes to be mis-spliced: calcium/calmodulin-dependent kinase II delta, cardiac troponin T, and Cypher. Cardiomyocytes deficient in ASF/SF2 display a hypercontractile phenotype due to a defect in postnatal splicing switch of CaMKIIδ. This failure results in mis-targeting of the kinase to sarcolemmal membranes, causing severe excitation-contraction coupling defects.

SRp38 (also known as SRSF10) null mice were embryonically lethal due to cardiac defects including atrial and ventricular septal defects. Furthermore, knockout of SRp38 resulted in mis-splicing of triadin, a cardiac protein that functions in regulating calcium release from the sarcoplasmatic reticulum during excitation-contraction coupling (Feng et al. 2009).

One of the few muscle-specific splicing factors RNA-binding motif protein 24 (Rbm24) was recently shown to be a major regulator of heart and skeletal muscle splicing (Yang et al. 2014). Rbm24 knockout mice were lethal and died between E12.5 and E14.5 showing multiple cardiac malformations, including ventricular septum defect, reduced trabeculation and compaction, and dilated atria. Strikingly, the formation of sarcomeres was almost completely absent in cardiomyocytes. This suggests a crucial role for Rbm24 in sarcomerogenesis, which was in line with a previously published zebrafish model where rbm24 was knocked down using morpholinos (Poon et al. 2012). Sixty-eight Rbm24-dependent splicing events were identified, of which most genes have a previously described critical role in cardiac development, cardiomyopathy, and sarcomerogenesis (Yang et al. 2014). Furthermore, the majority of the alternative splicing events was exon exclusions, which indicates that Rbm24 is a splicing activator.

A more recent study demonstrates a novel molecular mechanism whereby hypoxia-induced upregulation of the splicing factor SF3B1 (Splicing factor 3B subunit 1) causes mis-splicing of ketohexokinase and triggers the onset of cardiac hypertrophy by enforcing fructolysis (Mirtschink et al. 2015). Interestingly, cardiomyocyte-specific ablation of SF3B1 or ketohexokinase prevents the metabolic switch and protects from pathological cardiac growth.

Members of the FOX-protein family are also dysregulated in heart disease. Downregulation of RBFOX1 (RNA-binding protein, fox-1 homologue) is associated with heart failure in humans and mouse models, and the loss of Rbfox1 exacerbates pressure overload-induced heart failure in mice (Gao et al. 2016). It was shown that Rbfox1 controls the splicing of the myocyte enhancer-2 (Mef2) family members by regulating the splicing of the mutually exclusive exons α1 and α2, which interferes with the transcriptional activity of Mef2 family members. Finally, induction of Rbfox1 expression in murine pressure overload models substantially attenuated cardiac hypertrophy and progression to heart failure (Gao et al. 2016).

Expression of Rbfox2 is also decreased in the pressure-overloaded mouse heart, and conditional deletion of Rbfox2 leads to dilated cardiomyopathy and heart failure (Wei et al. 2015). Splicing analysis of both pressure-overloaded hearts and Rbfox2 knockout hearts revealed enrichment in developmentally regulated splicing events.

Altogether, the splicing factors described above seem to each control the alternative splicing of a specific subset of genes, which when disturbed leads to cardiac defects or a cardiomyopathy phenotype. It would therefore be interesting to start investigating the clinical relevance of these splicing factors by including them in routine genetic screens for familial cardiomyopathies.

### Splicing factors in human cardiomyopathies

One of the best-known splicing-associated diseases is myotonic dystrophy, which is a neuromuscular disease characterised by dilated cardiomyopathy, cardiac conduction defects, and skeletal muscle weakness (Liquori et al. 2001; Pelargonio et al. 2002). Type I myotonic dystrophy (DM1) is caused by a mutational expansion of a repetitive trinucleotide sequence (CUG) in the 3′-untranslated region of the DMPK gene (myotonic dystrophy protein kinase gene). Generally, 5–34 CUG repeats are observed in normal alleles but their number reaches 50–2000 in DM1. The less frequent type 2 myotonic dystrophy (DM2) is caused by CCUG expansion in an intron of the zinc finger protein 9 (ZFN9) gene.

In DM1, the widespread alternative splicing changes are a result of the CUG expansions that act as a molecular sponge for the MBNL splicing factors (Philips et al. 1998). These mutant RNAs alter the activities of RNA processing factors, including MBNL proteins, leading to re-expression of foetal isoforms in adult tissues and DM1 pathology (Fardaei et al. 2002; Thomas et al. 2017).

To date, there is only one splicing factor that has been identified as a direct cause of cardiomyopathy. Mutations in RNA-binding motif protein 20 (RBM20) were shown to cause an early onset and clinically aggressive form of DCM (Beqqali et al. 2016; Brauch et al. 2009; Li et al. 2010; Refaat et al. 2012). Next-generation sequencing in a large cohort of idiopathic DCM (iDCM) patients revealed that titin (TTN) is the most frequently affected gene in DCM. Interestingly, RBM20 was found among the most frequently mutated genes in DCM (Haas et al. 2014). Studies in rodents demonstrated that RBM20 is highly enriched in the heart and regulates the alternative splicing of a set of genes as a splicing repressor of which titin (TTN) is its most prominent splicing target (Dauksaite and Gotthardt 2018; Guo et al. 2012; Li et al. 2013; Maatz et al. 2014).

TTN is a giant sarcomeric protein, which acts as a molecular spring in the sarcomere, and as such, defines the passive stiffness of the cardiomyocyte. Titin-based passive stiffness is mainly adjusted by isoform switching through alternative splicing between the longer titin N2BA isoform and the N2B isoform. A perinatal switch in titin isoforms from the foetal compliant titin N2BA to the less compliant (stiffer) N2B adult isoform occurs in the heart to adapt to the postnatal cardiac load demands (Opitz et al. 2004).

Altered splicing of *TTN* occurs in a number of cardiac diseases such as heart failure, ischemic heart disease, and hypertrophic cardiomyopathy (Chauveau et al. 2014). Studies have shown a shift in expression from the stiff N2B isoform of titin towards the compliant N2BA isoform in human cardiomyopathies. This shift has been associated with reduced myofibrillar stiffness in DCM patients (Makarenko et al. 2004; Nagueh et al. 2004), which has been proposed as a mechanism to improve diastolic filling (Fukuda et al. 2003; Methawasin et al. 2014). In addition, an increase in compliant titin has also been suggested to impair systolic performance by affecting the Frank-Starling mechanism (FSM), i.e., the ability of the sarcomere to increase contractile force in response to stretch (Beqqali et al. 2016; Methawasin et al. 2014).

Guo and colleagues were the first to demonstrate that Rbm20 is a major regulator of titin alternative splicing (Guo et al. 2012). Loss of Rbm20 leads to aberrant inclusion of many exons in the TTN transcript, resulting in the expression of very large and compliant TTN isoforms in the heart, which is believed to underlie the DCM phenotype in RBM20 mutation carriers.

The same group has proposed that regulating titin splicing, by means of modulating Rbm20 levels, could be beneficial for the heart in the setting of heart failure with preserved ejection fraction (HFpEF) (Guo and Sun 2018; Methawasin et al. 2014, 2016). Although it may be advantageous to modulate Rbm20-dependent titin splicing to decrease passive stiffness in certain types of heart disease where passive stiffness is increased, the effect on other Rbm20 targets such as calcium handling genes must be carefully evaluated.

A heterozygous loss of Rbm20 in mice is sufficient to induce a shift in CamkIIδ isoforms, which leads to a disturbed calcium handling in cardiomyocytes (van den Hoogenhof et al. 2018). Importantly, patients with mutations in RBM20 often suffer from lethal arrhythmias that cannot be explained by mis-splicing of titin alone. In addition to adapting titin isoform expression and thus cardiac filling in diastole, RBM20 affects a set of at least 30 genes, which have been implied in diastolic function, sarcomere assembly, and ion transport. These genes include sarcomeric genes such myomesin 1, but also Ca^2+^ and ion handling genes such as calcium/calmodulin kinase IIδ (Camk2d, ryanodine receptor 2 (Ryr2) and calcium voltage-gated channel subunit alpha 1C (Cacna1c) (Guo et al. 2012; Maatz et al. 2014). Aberrant splicing of CamkIIδ in Rbm20 KO mice results in a remarkable shift of CamkIIδ towards the δ-A isoform that is known to activate the L-type Ca^2+^ channel. In line with this, an increased L-type calcium current, intracellular Ca^2+^ overload and increased sarcoplasmic reticulum (SR) Ca^2+^ content was found in Rbm20-depleted myocytes (van den Hoogenhof et al. 2018). Therefore, the proposed modulation of Rbm20 levels by Guo and colleagues in the setting of HFpEF is likely to affect calcium handling (and other important processes) in cardiomyocytes and lead to undesirable arrhythmias.

Intriguingly, RBM20 was also shown to play a role in the formation of circular RNAs from the titin gene. It was hypothesised that RBM20 is crucial for the formation of a subset of circRNAs that originate from the I band of the titin gene. Furthermore, by excluding specific exons from the pre-mRNA, RBM20 provides the substrate to form this class of RBM20-dependent circRNAs (Aufiero et al. 2018; Khan et al. 2016). It would be interesting to investigate the function of these circular RNAs and their possible role in cardiomyopathy.

In-depth reviews about the role of RBM20 in cardiomyopathy were recently published elsewhere (Ma et al. 2016; Rexiati et al. 2018).

## Conclusion and future perspectives

In the past decade, it has become clear that alternative splicing is a tightly regulated process in the heart which when disturbed leads to a variety of cardiomyopathies. Whole transcriptome analysis by microarray and RNA sequencing has revealed that the heart undergoes a critical perinatal switch from foetal to adult splicing programme, which is reactivated upon pathological hypertrophy. Although the reasons and molecular mechanisms underlying the reactivation of the splicing programme are unclear, it is thought that a general downregulation of important splicing factors like RBFOX1 and MBNL is contributing to this. Whether this downregulation of splicing factors is mediated by transcriptional mechanisms or post-transcriptional mechanisms such as miRNAs is yet to be determined. Further research is needed to gain more insight into the contribution of mis-splicing to the progression to heart failure and to develop strategies to reverse this maladaptive process.

The recent reports on the functional contribution of mis-spliced individual protein-coding genes in cardiomyopathies such as titin and Camk2d are only small pieces of the puzzle, as the majority of the transcriptome is comprised of non-coding RNAs that are subject of extensive alternative splicing.

The functional consequences of alternative splicing in non-coding RNAs, such as circular RNAs, remain to be investigated. Unravelling the biogenesis, regulation, and function of circRNAs in the heart will likely open a major new field in molecular cardiology in the coming decade.

Although the functional consequences of mis-spliced genes needs more research, the observation of specific splicing signatures by itself can be very informative. It is of clinical relevance to further investigate the potential of splicing profiles as highly specific diagnostic and prognostic biomarkers of cardiomyopathy.

It is now believed that up to 60% of disease causing-mutations influence alternative splicing (Lopez-Bigas et al. 2005; Pagani and Baralle 2004). However, RBM20 is the only identified splicing factor to be mutated in human cardiomyopathy. It is not known yet how many other RNA-binding proteins are involved in splicing control in the heart, and we expect that there are many more (alternative) splicing factors to be discovered. It would therefore be interesting to design unbiased approaches to identify the RNA-bound proteome of the heart in different stages of development and disease. This will allow us to identify novel critical post-transcriptional regulators that could be of clinical relevance. Utilising crosslinking RNA immunoprecipitation methods combined with LC-MS proteomics techniques can achieve this. Furthermore, it would be helpful to start routine genetic screening for mutations in newly identified splicing factors like RBM24 and RBFOX1 to investigate their role in human cardiomyopathy.

Therapeutic strategies are currently being developed to rescue alternative splicing defects in several human diseases such as use of antisense oligonucleotides (AONs).

AONs are designed to bind to a specific splicing RNA sequence to manipulate splicing. Duchenne’s muscular dystrophy (DMD), caused by mutations in the dystrophin gene, is the first disease in which AONs have been clinically tested. The administration of AONs to DMD patients promoted exon skipping of the mutated exon (to avoid premature truncation of the protein) and modest improvements in exercise capacity (Goemans et al. 2011).

Other AON strategies have been tested in preclinical models of disease including progeria (LMNA gene) (Scaffidi and Misteli 2005), spinal muscular atrophy (SMN2) (Hua et al. 2011), and myotonic dystrophy (DMPK) (Wheeler et al. 2012). Although these strategies showed positive results, they still need to be refined and more efficient as the beneficial effects of these therapies remain modest.

Another strategy may involve re-introducing splicing factors by viral means to restore appropriate alternative splicing as many cardiomyopathies are characterised by a downregulation of splicing factors at the protein level.

Although therapeutic strategies are underway, further insights into the molecular mechanisms of cardiac alternative splicing are necessary to eventually enable us to manipulate alternative splicing in the benefit of the patient.
